# Supplementary material for: Functional specialization within the inferior parietal lobes across cognitive domains
Source: eLife. 2021 Mar 2;10:e63591. doi: 10.7554/eLife.63591 (PMC7946436; doi:10.7554/eLife.63591)
Supplement: Supplementary file 1. [file elife-63591-supp1.docx]

**Table S1**: Mass-univariate activations peaks for the three functional domains.

| **Region** | | **Anatomical assignment** | | **Hemis- phere** | **MNI coordinates  X Y Z** | | | **T-value** | **Cluster size** |
| --- | --- | --- | --- | --- | --- | --- | --- | --- | --- |
| Attentional reorienting | |  |  | |  |  |  |  |  |
|  | Supramarginal gyrus | PFm | R | | 57 | -48 | 24 | 9.68 | 181 |
|  | Superior temporal gyrus /   angular gyrus | PGa | R | | 63 | -45 | 17 | 9.23 |  |
|  | Middle temporal gyrus /   angular gyrus | PGp | R | | 48 | -63 | 14 | 8.42 |  |
|  | Middle temporal gyrus |  | R | | 57 | -57 | -1 | 7.03 |  |
|  | Precuneus | 7P | L | | 3 | -54 | 52 | 9.46 | 123 |
|  | Precuneus | 5L | R | | 6 | -60 | 59 | 8.27 |  |
|  | Precuneus | 7A | L | | 12 | -66 | 52 | 7.21 |  |
|  | Precentral gyrus |  | L | | -27 | -3 | 59 | 9.32 | 54 |
|  | Superior frontal gyrus |  | R | | 30 | -6 | 63 | 8.36 | 34 |
|  | Middle frontal gyrus |  | R | | 36 | -3 | 56 | 7.33 |  |
|  | Precentral gyrus |  | R | | 36 | 3 | 49 | 7.00 |  |
|  | Inferior parietal lobe | PF | L | | -54 | -42 | 38 | 7.35 | 33 |
|  | Postcentral gyrus |  | L | | -45 | -33 | 49 | 7.04 |  |
|  | Inferior parietal lobule | hIP2 | L | | -45 | -39 | 42 | 6.99 |  |
|  | Superior parietal lobe | hIP1 | R | | 30 | -48 | 42 | 10.10 | 31 |
| Lexical decisions | |  |  | |  |  |  |  |  |
|  | Angular gyrus | PGp | L | | -51 | -72 | 28 | 8.00 | 73 |
|  | Angular gyrus | PGp | L | | -39 | -75 | 42 | 7.36 |  |
|  | Angular gyrus | PGa | L | | -45 | -57 | 35 | 7.22 |  |
|  | Middle cingulate gyrus | 5M | L | | -6 | -30 | 42 | 9.07 | 50 |
|  | Superior frontal gyrus |  | L | | -18 | 36 | 49 | 7.94 | 34 |
|  | Middle frontal gyrus |  | L | | -27 | 24 | 52 | 6.64 |  |
| Perspective taking | |  |  | |  |  |  |  |  |
|  | Supplementary motor cortex |  | L | | -3 | 15 | 52 | 5.15 | 184 |
|  | Superior medial gyrus |  | L | | -6 | 24 | 42 | 4.37 |  |
|  | Superior frontal gyrus |  | L | | -15 | 12 | 63 | 3.84 |  |
|  | Posterior medial frontal g. |  | R | | 12 | 6 | 52 | 3.73 |  |
|  | Inferior frontal gyrus | BA45 | L | | -45 | 24 | 31 | 5.11 | 59 |
|  | Precentral gyrus |  | L | | -39 | 3 | 42 | 4.77 | 45 |
|  | not assigned |  | L | | -45 | 6 | 56 | 3.84 |  |
|  | Precentral gyrus | BA44 | L | | -48 | 9 | 35 | 3.81 |  |
|  | Middle frontal gyrus |  | L | | -42 | 3 | 52 | 3.72 |  |
|  | Angular gyrus | PGa | R | | 51 | -60 | 31 | 5.15 | 32 |
|  | Precuneus |  | R | |  |  |  | 4.50 | 31 |
|  | Precuneus |  | L | |  |  |  | 3.98 |  |
|  | Middle temporal gyrus |  | R | | 54 | -18 | -15 | 4.68 | 20 |
|  | Middle temporal gyrus |  | R | | 60 | -15 | -11 | 4.29 |  |

*Note: Attention and semantic tasks: thresholded at p=0.05, FWE corrected, cluster extent ≥20 voxels. Social cognition task: thresholded at p=0.001, uncorrected, cluster extent ≥20 voxels. Anatomical assignment according to SPM Anatomy toolbox (v. 22c).*
