# Supplementary material for: Functional specialization within the inferior parietal lobes across cognitive domains
Source: eLife. 2021 Mar 2;10:e63591. doi: 10.7554/eLife.63591 (PMC7946436; doi:10.7554/eLife.63591)
Supplement: Supplementary file 2. [file elife-63591-supp2.docx]

**Table S2**: Intrinsic effective connectivity and task-specific self-connectivity modulation

| **Source subregion** |  | **Target subregion** | **Strength** | **95%-CI** |
| --- | --- | --- | --- | --- |
| **Intrinsic connectivity** | | | | |
| L-ant | → | L-ant | -1.20 | [-1.21; -1.20] |
| L-ant | → | L-post | -0.37 | [-0.38; -0.37] |
| L-ant | → | R-ant | -0.33 | [-0.33; -0.33] |
| L-ant | → | R-post | -0.56 | [-0.56; -0.56] |
| L-post | → | L-ant | -0.05 | [-0.06; -0.05] |
| L-post | → | L-post | -0.79 | [-0.81; -0.79] |
| L-post | → | R-ant | 0.15 | [0.15; 0.16] |
| L-post | → | R-post | -0.29 | [-0.29; -0.29] |
| R-ant | → | L-ant | -0.27 | [-0.27; -0.27] |
| R-ant | → | L-post | 0.38 | [0.39; 0.39] |
| R-ant | → | R-ant | -1.13 | [-1.14; -1.13] |
| R-ant | → | R-post | 0.87 | [0.87; 0.88] |
| R-post | → | L-ant | 0.34 | [0.34; 0.35] |
| R-post | → | L-post | 0.42 | [0.42; 0.43] |
| R-post | → | R-ant | -0.30 | [-0.30; -0.30] |
| R-post | → | R-post | -1.14 | [-1.15; -1.14] |
|  |  |  |  |  |
| **Source subregion** |  | **Target subregion** | **Modulation strength** | **p-value** |
| **Self connectivity** | | | | |
| Semantics | | | | |
| L-ant | → | L-ant | -5.53 | 0.0006 |
| L-post | → | L-post | -6.88 | 0.0010 |
| Social Cognition | | | | |
| R-ant | → | R-ant | -4.01 | 0.0055 |

*Note: Intrinsic connectivity parameters (‘A-matrix’) and significant modulatory parameters (‘B-matrix’,* α ≤ 0.01*) of self-connections. Strength is given as posterior expectation from the optimum Bayesian parameter average model. The 95% confidence intervals were built from the corresponding Bayesian parameter covariances and exceed zero for all parameters. P-values for self-connectivity are based on a random effects permutation test for the null hypothesis ‘no parameter difference between tasks’.*
