# Supplementary material for: Functional specialization within the inferior parietal lobes across cognitive domains
Source: eLife. 2021 Mar 2;10:e63591. doi: 10.7554/eLife.63591 (PMC7946436; doi:10.7554/eLife.63591)
Supplement: Transparent reporting form [file elife-63591-transrepform.pdf]

eLIFE
1st Floor
24 Hills Road
Cambridge CB2 1JP, UK
P 01223 855340
W elifesciences.org
T @elife

eLife's transparent reporting form

We encourage authors to provide detailed information *within their submission* to facilitate the interpretation and replication of experiments. Authors can upload supporting documentation to indicate the use of appropriate reporting guidelines for health-related research (see EQUATOR Network), life science research (see the BioSharing Information Resource), or the ARRIVE guidelines for reporting work involving animal research. Where applicable, authors should refer to any relevant reporting standards documents in this form.

If you have any questions, please consult our Journal Policies and/or contact us: editorial@elifesciences.org.

Sample-size estimation

- You should state whether an appropriate sample size was computed when the study was being designed
- You should state the statistical method of sample size computation and any required assumptions
- If no explicit power analysis was used, you should describe how you decided what sample (replicate) size (number) to use

Please outline where this information can be found within the submission (e.g., sections or figure legends), or explain why this information doesn't apply to your submission:

In this functional magnetic resonance imaging study, we utilized three well-established tasks to probe the three cognitive domains under investigation and their relation to each other. Accordingly, we chose the sample size to surpass the typical sample size that has been used in past studies in the respective scientific fields. Additionally, all subjects underwent several sessions (Materials and Methods -> Experimental Design, p. 24).

Replicates

- You should report how often each experiment was performed
- You should include a definition of biological versus technical replication
- The data obtained should be provided and sufficient information should be provided to indicate the number of independent biological and/or technical replicates
- If you encountered any outliers, you should describe how these were handled
- Criteria for exclusion/inclusion of data should be clearly stated
- High-throughput sequence data should be uploaded before submission, with a private link for reviewers provided (these are available from both GEO and ArrayExpress)

Please outline where this information can be found within the submission (e.g., sections or figure legends), or explain why this information doesn't apply to your submission:

eLife Sciences Publications, Ltd is a limited liability non-profit non-stock corporation incorporated in the State of Delaware, USA, with company number 5030732, and is registered in the UK with company number FC030576 and branch number BR015634 at the address 1st Floor, 24 Hills Road, Cambridge CB2 1JP | August 2014

The experimental design with its repetitive and independent acquisition strategy is detailed under Materials and Methods -> Experimental Design (p. 24).
No subjects were excluded from the analyses.
Information on single volume masking via framewise displacement regressors of the fMRI data: Materials and Methods -> Specification of the general linear models (p. 28)
Exclusion of single trials due to wrong responses: Materials and Methods -> Task-predictive information of the IPL subregions (p. 30); and Materials and Methods -> Specification of the general linear models (p. 28).

eLife Sciences Publications, Ltd is a limited liability non-profit non-stock corporation incorporated in the State of Delaware, USA, with company number 5030732, and is registered in the UK with company number FC030576 and branch number BR015634 at the address 1st Floor, 24 Hills Road, Cambridge CB2 1JP | August 2014

Statistical reporting

- Statistical analysis methods should be described and justified
- Raw data should be presented in figures whenever informative to do so (typically when N per group is less than 10)
- For each experiment, you should identify the statistical tests used, exact values of N, definitions of center, methods of multiple test correction, and dispersion and precision measures (e.g., mean, median, SD, SEM, confidence intervals; and, for the major substantive results, a measure of effect size (e.g., Pearson's r, Cohen's d)
- Report exact p-values wherever possible alongside the summary statistics and 95% confidence intervals. These should be reported for all key questions and not only when the p-value is less than 0.05.

Please outline where this information can be found within the submission (e.g., sections or figure legends), or explain why this information doesn't apply to your submission:

The statistical methods used are described in detail in the Materials and Methods section (pp. 24) and information on the initial standard analysis of the acquired fMRI data is given under 'Materials and Methods -> Specification of the general linear models' (p. 28). Detailed results of this initial mass-univariate analysis and information of multiple test corrections are shown accordingly ('Results -> IPL recruitment in different cognitive tasks: neural activity responses' (pp. 6) and Table S1).
Behavioral results with single subject data and summary statistics: Figure 1-figure supplement 1
Details on the predictive analyses, including accuracy metrics, chance levels, and cross-validation schemes are presented under 'Results -> Task activity responses are predictive for different cognitive domains' (p. 10) and in Figure 3 (p. 11). Because this method is of a predictive nature and not from the null-hypothesis testing domain, p-values do not apply.
The non-parametric permutation test utilized for the functional connectivity analyses is detailed under 'Materials and Methods -> Task-evoked functional connectivity shifts' (p. 31) and significant results are shown in Figure 4 (p. 13).
Details on the dynamic causal modeling analysis are provided (Materials and Methods -> Task-specific effective connectivity modulation (p. 32)) and exact p-values are reported in Table 2 (p. 17) and Table S2.

(For large datasets, or papers with a very large number of statistical tests, you may upload a single table file with tests, Ns, etc., with reference to sections in the manuscript.)

Group allocation

- Indicate how samples were allocated into experimental groups (in the case of clinical studies, please specify allocation to treatment method); if randomization was used, please also state if restricted randomization was applied
- Indicate if masking was used during group allocation, data collection and/or data analysis

Please outline where this information can be found within the submission (e.g., sections or figure legends), or explain why this information doesn't apply to your submission:

Methods -> Experimental design

Additional data files ("source data")

- We encourage you to upload relevant additional data files, such as numerical data that are represented as a graph in a figure, or as a summary table
- Where provided, these should be in the most useful format, and they can be uploaded as "Source data" files linked to a main figure or table
- Include model definition files including the full list of parameters used
- Include code used for data analysis (e.g., R, MatLab)
- Avoid stating that data files are "available upon request"

Please indicate the figures or tables for which source data files have been provided:

Data and Code Availability statements are provided (p. 33).
